# Mesopore Formation and Silicon Surface Nanostructuration by Metal-Assisted Chemical Etching With Silver Nanoparticles

**DOI:** 10.3389/fchem.2020.00658

**Published:** 2020-07-30

**Authors:** Elisa Pinna, Sylvain Le Gall, Encarnacion Torralba, Guido Mula, Christine Cachet-Vivier, Stéphane Bastide

**Affiliations:** ^1^PoroSiLab, Dipartimento di Fisica, Università degli Studi di Cagliari, Monserrato, Italy; ^2^Group of Electrical Engineering of Paris (GeePs), CNRS, Univ. Paris-Saclay, CentraleSupélec, Sorbonne Univ., Gif-sur-Yvette, France; ^3^CNRS, Univ Paris Est Creteil, ICMPE, UMR7182, Thiais, France

**Keywords:** MACE, silver nanoparticle, black silicon, reflectivity, texturization, nanostructuration

## Abstract

This article presents a study on Metal-Assisted Chemical Etching (MACE) of silicon in HF-H_2_O_2_ using silver nanoparticles as catalysts. Our aim is a better understanding of the process to elaborate new 3D submicrometric surface structures useful for light management. We investigated MACE over the whole range of silicon doping, i.e., p^++^, p^+^, p, p^−^, n, n^+^, and n^++^. We discovered that, instead of the well-defined and straight mesopores obtained in p and n-type silicon, in p^++^ and n^++^ silicon MACE leads to the formation of cone-shaped macropores filled with porous silicon. We account for the transition between these two pore-formation regimes (straight and cone-shaped pores) by modeling (at equilibrium and under polarization) the Ag/Si/electrolyte (HF) system. The model simulates the system as two nanodiodes in series. We show that delocalized MACE is explained by a large tunnel current contribution for the p-Si/Ag and n-Si/HF diodes under reverse polarization, which increases with the doping level and when the size of the nanocontacts (Ag, HF) decreases. By analogy with the results obtained on heavily doped silicon, we finally present a method to form size-controlled cone-shaped macropores in p silicon with silver nanoparticles. This shape, instead of the usual straight mesopores, is obtained by applying an external anodic polarization during MACE. Two methods are shown to be effective for the control of the macropore cone angle: one by adjusting the potential applied during MACE, the other by changing the H_2_O_2_ concentration. Under appropriate etching conditions, the obtained macropores exhibit optical properties (reflectivity ~3 %) similar to that of black silicon.

## Introduction

Metal-assisted chemical etching (MACE) of silicon is a powerful technique to produce surface nanostructures with high aspect ratios. Networks of nanowires, nanopores or nanocones and more complex patterns synthesized in this way find applications in various fields like microelectronics, solar energy conversion or chemical/biological sensing.

Recently, the texturing at the submicrometer scale of silicon into so called “black silicon” has been the object of a renewed interest for crystalline silicon solar cells (Otto et al., 2015). This is largely due to advances in surface passivation, notably with the optimization of the emitter doping to avoid Auger recombination (Oh et al., 2012) and the use of thin passivating dielectric layers (e.g., Al_2_O_3_) to overcome surface recombination involved with large surface areas (Savin et al., 2015). Thus, “black silicon” is being considered for practical photovoltaic applications since it offers a much lower reflectivity than conventional surfaces (e.g., pyramids with sizes of several micrometers), without the need of antireflection coating, and is also very efficient for light trapping in the cell. However, an adequate three-dimensional (3D) control of the texture is essential to achieve the required efficiency of the cells.

Within this framework, MACE has been shown to be a method of choice to produce efficient nanostructures for light management in silicon solar cells (Koynov et al., 2006). It continues to be studied as it is a relatively easy chemical method to implement and uses simple reagents. Its potential has not yet been fully explored, far from it, as many parameters have a relevant impact on the etching process. As a result, current research is devoted both to understanding the mechanisms of MACE and to determining etching conditions and modalities to fabricate tailored surface structures for efficient light coupling.

The nature of the metal is of primary importance in MACE. Silver nanoparticles are known to dig well-defined straight mesopores whose walls are smooth and with diameters set by the nanoparticle sizes (Tsujino and Matsumura, 2005). Silver is therefore used for highly localized etching and the obtention of well-resolved nanostructures. On the contrary, gold or platinum nanoparticles give rise to the formation of mesopores surrounded by a cone-shaped volume of porous silicon (Lee et al., 2008). The reason for this difference is the nature of the Schottky metal/Si junction, which can be rectifying (silver) or ohmic (gold, platinum). In the first case (i.e., rectifying junction), the injection of holes is not possible while in the second it causes the polarization of the bulk and thus leads to the formation of porous silicon at the mesopore Si/electrolyte (HF) interface (Torralba et al., 2016).

Other factors are also important in MACE, such as the doping of the silicon substrate, the metal shape (e.g., nanoparticle or mesh) or the composition of the etching solution. Under certain etching conditions, silver nanoparticles or silver-meshes have been shown to lead to the formation of porous silicon, i.e., to a delocalized rather than localized etching, in contrast to what is usually observed (Chartier et al., 2008; Zhang et al., 2008; Geyer et al., 2013). The influence of substrate doping on the formation of mesopores by MACE with silver nanoparticles has therefore not been fully established yet.

In this work, we have studied the MACE process in view of an improved understanding for the control of the surface structures 3D shapes. In particular, the formation of mesopores in silicon with silver nanoparticles in HF/H_2_O_2_ was studied on a wide range of substrate dopings, namely p^++^, p^+^, p, p^−^, n, n^+^, and n^++^. Our most significant discovery is that, instead of the well-defined nanometer-sized mesopores with smooth walls obtained in p-type and n-type silicon, a MACE process based on silver nanoparticles in highly doped p- and n-type silicon leads to the formation of micrometer-sized cone-shaped pores filled with porous silicon. We try to account for the transition between these two pore formation regimes by modeling the Ag/Si/electrolyte interface at the nanoscale, at equilibrium and under etching conditions (electrostatic polarization).

Finally, we present results on the effect of adding an external polarization during MACE of p-type silicon with silver nanoparticles as a method to form cone-shaped macropores rather than the usual straight mesopores. We test how the cone angle can be controlled by tuning the applied potential or the concentration of H_2_O_2_ and the effect it has on the surface reflectivity.

## Materials and Methods

### Silicon Substrates

Polished crystalline (100) p-type (boron) and n-type (phosphorus) silicon wafers (300–500 μm in thickness) from Sil'tronix and ITME were used, with three resistivities for each doping: n^++^ (~2.5 10^−3^ Ω cm), n^+^ (~3.0 10^−2^ Ω cm), n (~2.0 Ω cm), p (~2.0 Ω cm), p^+^ (8.0 10^−2^ Ω cm), p^++^ (~3.5 10^−3^ Ω cm), and in addition p^−^ doped silicon with a resistivity of ~10 Ω cm.

### Chemicals

Analytical grade (VWR chemicals) 30 wt.% H_2_O_2_, 96 wt.% H_2_SO_4_, 40 wt.% HF, 65 wt.% HNO_3_ and ultra-pure water (18.2 MΩ cm, Millipore) were used in the all experiments. The AgNO_3_ salt (VWR) was 99 wt.%.

### Silver Deposition

Before silver deposition and etching, the silicon wafers were cleaved into 1.2 × 1.2 cm^2^ pieces, cleaned in H_2_SO_4_-H_2_O_2_ (3:1) and rinsed with ultra-pure water.

In order to obtain silver nanoparticles at the surface of the silicon substrate, a 10 nm thick silver layer was deposited using a Cressinton 208HR sputter coater and subsequently annealed 20 min at 275°C under Ar atmosphere. The nanoparticle size distribution was established from SEM observations and ImageJ analysis (*cf*. Figure A in Supplementary Information). In some cases, silver nanoparticles were deposited chemically by dipping the silicon samples in a solution of AgNO_3_ (1 mmol L^−1^) - HF (0.14 mol L^−1^) during 1 min (*cf*. Supplementary Information).

### MACE and MACE Under Electrochemical Polarization

MACE was performed by dipping the samples in an aqueous solution of 1.21 mol L^−1^ HF and 0.21 mol L^−1^ H_2_O_2_ for 20 min in all cases. The molar ratio ρ of the solution, i.e., [HF]/([HF] + [H_2_O_2_]), is equal to 0.85.

All MACE experiments under electrochemical polarization were performed in a home-made O-ring three-electrode cell. The silicon substrates (with or without silver nanoparticles) were used as working electrode and a Pt wire as counter electrode. A K_2_SO_4_ saturated Hg/Hg_2_SO_4_ electrode (SME), protected from the HF solution by a KCl saturated agar-agar bridge, was used as reference. The geometrical area of the Si/electrolyte contact was 0.38 cm^2^ (delimited by an O-ring). The silicon back contact was made by rubbing InGa. No supporting electrolyte was added to the HF-H_2_O_2_ solution (20 mL).

Given the negligible oxidizing effect of O_2_ in presence of H_2_O_2_, the working solutions were not degassed for open circuit potential (OCP) measurements and MACE experiments.

### Instrumentation

Scanning electron microscopy (SEM) images and Energy Dispersive X-ray Spectroscopy (EDS) were obtained with a Merlin FEG microscope from Zeiss equipped with AZtec systems (EDS Advanced, HKL Advanced Nordlys Nano, Oxford Instruments).

Cyclic voltammetry and chronoamperometry were performed with a PGSTAT 20 potentiostat/galvanostat Metrohm Autolab, equipped with Nova software.

The optical reflectivity measurements were performed in the 350–1500 nm range using a Hitachi UV-VIS-NIR 4001 spectrophotometer fitted with an integrating sphere.

### Modeling

Numerical simulations in 2D of the valence and conduction band modulation at the Ag/Si/electrolyte interfaces were performed using the commercial TCAD software (Atlas from Silvaco, *cf*. Torralba et al., 2016) based on a finite volume method. This simulator solves the physical equations governing the electrostatics (Poisson, electro-neutrality) and the transport of e^−^ and h^+^ (drift-diffusion) self-consistently on a 2D mesh.

The modeled structure is schemed in Figure B of Supplementary Information. It consists of a silicon substrate with a thickness of 100 μm and a width of 100 nm, with a 12 nm large silver pad surrounded by two electrolyte contacts. The electrolyte contacts are short-circuited (i.e., at same potential). The silver and electrolyte electrodes are separated by 1 nm of insulating vacuum to avoid charge transfer between them. The work functions of silicon are taken at 4.07 eV < W_Si_ < 4.29 eV depending on the doping level, W_Ag_ = 4.64 eV (Hölzl and Schulte, 1979), and W_El_ = 4.5 eV (determined in our experimental conditions, *cf*. Torralba et al., 2016 and its Supplementary Information). The Fermi level is set at 0 eV at the equilibrium. To mimic the MACE process, a positive polarization can be applied between the silver contact and the electrolyte.

## Results and Discussion

### Pore Morphology

**Figure 1:**
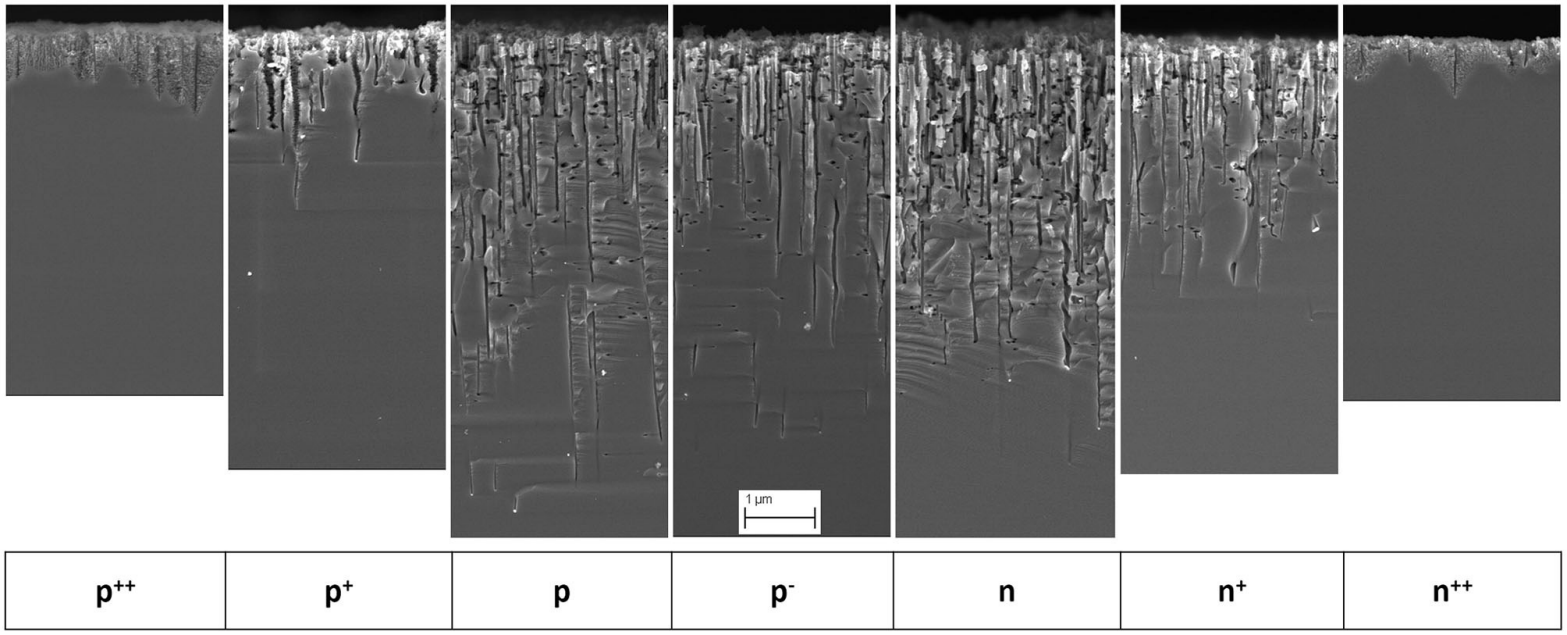
SEM images of mesopore tips formed by MACE with silver nanoparticles and HF-H_2_O_2_ in silicon substrates with doping levels: p^++^, p^+^, p, p^−^, n, n^+^, n^++^. Same magnification for all images. Scale bar: 1μm.

Figure 1 presents SEM images at the same magnification of mesopores obtained under identical MACE conditions of silicon substrates with doping levels ranging from p^++^ to n^++^.

**Figure 2:**
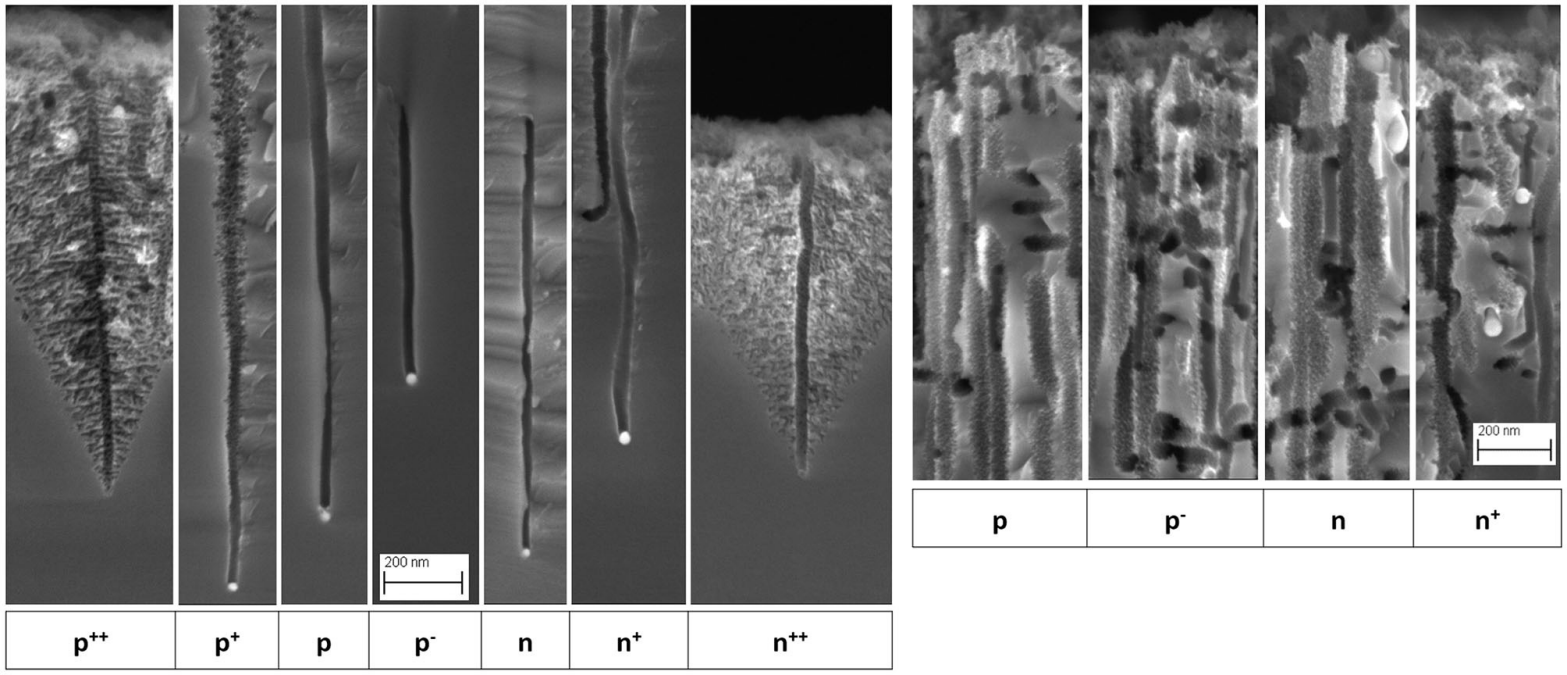
SEM images of mesopore tips (**left**) and tops (**right**), for the different substrate dopings. Same magnification for all images of each group. Note that the presence or absence of a nanoparticle at a pore tip is only a random result of cutting the sample for cross-sectional SEM imaging (depending on whether it remains stuck to the observed piece). Scale bar: 200 nm.

Figure 2 shows the pore tips (left) and tops (right) at higher SEM magnification.

In the case of p, p^−^, n and n^+^-type silicon, the tip sections exhibit a constant diameter and pore walls free of porous silicon. However, at the level of the surface, they all exhibit a certain widening and rough inner surfaces. Most of the pores are perpendicular to the surface but a significant number is also found parallel to the surface (Tsujino and Matsumura, 2007) have shown that silver nanoparticles create lateral pores because they can follow other preferential orientations equivalent to [100] [e.g., (010), (001)…] or when they have non-spherical shapes (pores are then dug in random or twisted directions). As a result, many silver nanoparticles are found close to the surface even after a long etching time. In the case of highly doped p^++^ and n^++^-type silicon, pores with a surrounding cone-shaped mesoporous silicon volume are formed. For p^+^-type silicon, mesoporous silicon is formed around the mesopores as well, but to a much lower extent. The pore length decreases strongly as the doping level increases, with a ratio of ~ 6–7 for p vs. p^++^ and n vs. n^++^.

These results are specific to the HF and H_2_O_2_ concentrations chosen for etching. For other HF and H_2_O_2_ concentrations, the frontiers in terms of doping level between cone-shaped pores (i.e., with mesoporous silicon) and straight pores formation may change. However, MACE with silver nanoparticles in low-doped (n- and p-type) silicon always leads to straight pores, irrespective of the reactant concentrations (for 0.7 < ρ < 1); this is reported for instance in Chartier *et al*. for p-type silicon (1-2 ohm.cm) in 14 mol L^−1^ HF and 3 mol L^−1^ H_2_O_2_ (i.e., ten times higher concentrations than in the present case).

In order to understand why and how the pore morphology changes with the type and level of doping, a modeling describing the silicon bands modulation around nanometer sized silver and electrolyte contacts has been carried out. This type of modeling has already provided insights into the photo/electrochemical behavior of metal decorated silicon electrodes (Nakato et al., 1988), on MACE (Kolasinski, 2016) and electrochemically assisted MACE mechanisms (Chourou et al., 2010; Huang et al., 2010; Torralba et al., 2016; Bastide et al., 2019). The results have also been interpreted in the light of data from the large literature existing on the electrochemistry of silicon in HF media, in particular the reference book by Lehmann (2002).

The common characteristic of MACE processes catalyzed by silver nanoparticles, irrespective of the silicon type and doping level, is the formation of a main pore having the dimensions and shape of the nanoparticle with nanometer-level accuracy. It is actually the basis for the formation of nanowires networks in AgNO_3_/HF solutions. This ultra-localized dissolution must correspond to a surface phenomenon where the silicon atoms located very close to the metal contact are subjected to a lateral electric field strong enough to attract their electrons directly to the metal.

The change in pore morphology is related to the additional presence of mesoporous silicon at the main pore walls. This can represent a thin layer of a few nanometers either along the pore (*cf*. p^+^ silicon in Figure 2) or just at its apex (*cf*. n^+^ silicon in Figure 2), or a cone of mesoporous silicon much wider than the main pore (*cf*. p^++^ and n^++^ silicon in Figure 2). This constitutes a delocalized dissolution implying a polarization of the Si/HF interface far away from the silver nanoparticle which is only possible through a polarization of the silicon bulk. It is this case that we can try to account the observed phenomena by a modeling of the process and from data in the literature.

### Band Bending Modeling

The modeled Ag/Si/electrolyte (HF) system consists in a silver pad on flat silicon surrounded by two electrolytic contacts (short-circuited) with a total width of 100 nm (*cf*. Figure B of Supplementary Information for details on the modeled device structure). The silver and electrolyte electrodes are separated by a gap of 1 nm, hence charge transfers only occurs through the Si/electrolyte (HF) and Si/Ag interfaces.

**Figure 3:**
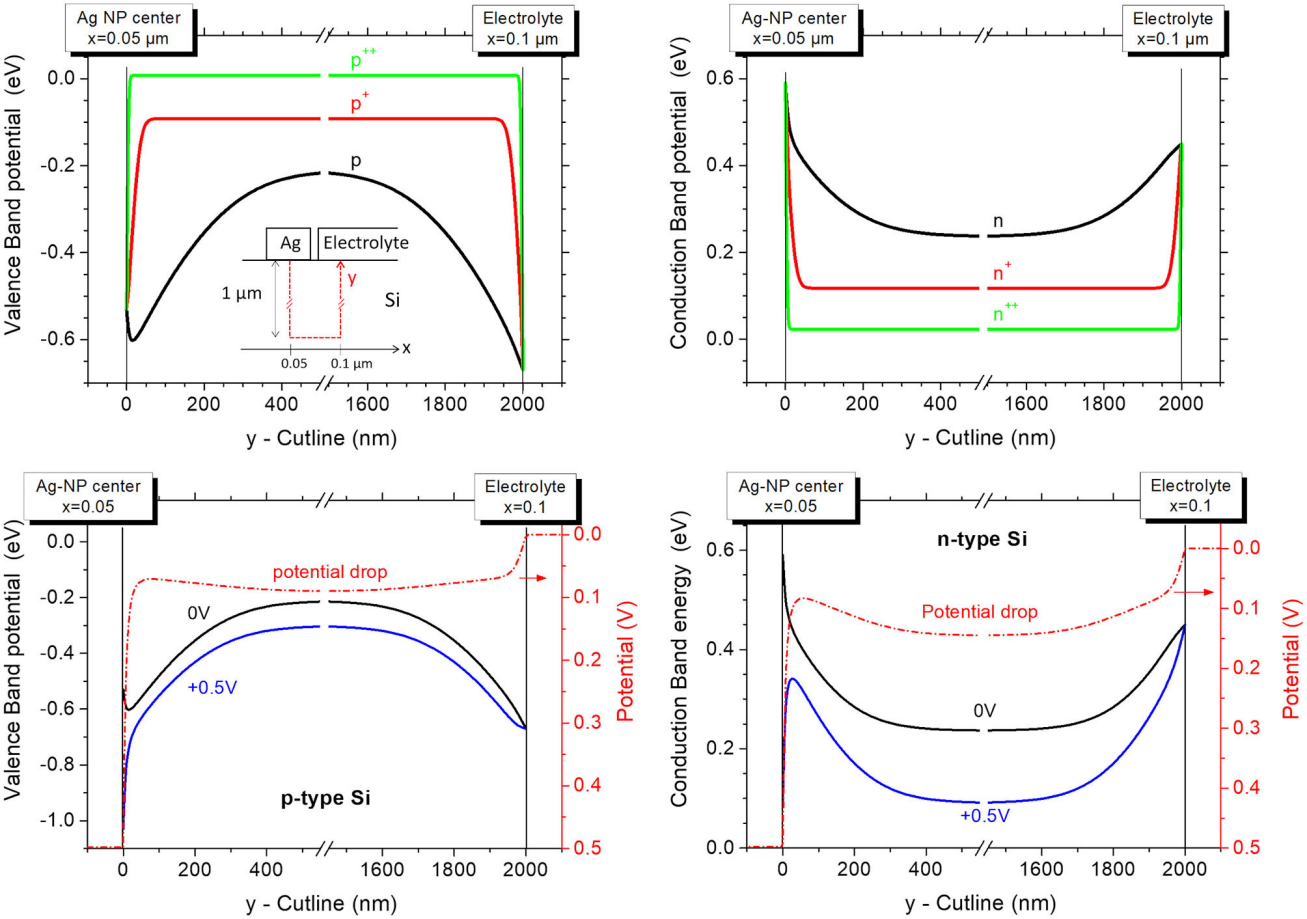
Band diagram (valence band and conduction band for p-type and n-type silicon, respectively) of the modeled Ag/Si/electrolyte (HF) system at equilibrium (top row) and under a positive polarization of silver vs. Electrolyte by +0.5 V (bottom row). x is the lateral position at the surface of the device and y the position along the cutline through bulk Si, as schematized in the upper-left band diagram and in Figure B of the Supplementary Information. Three doping levels for n and p-type silicon are represented (right and left column, respectively). The spatial distribution of the voltage drop is also presented (red dash-dot line).

Figure 3 presents the valence and conduction band diagrams for p-type and and n-type silicon at equilibrium (top row). They correspond to the band modulations along a cutline (*y*-coordinate) that connects the center of the silver nanoparticle to the electrolyte 50 nm away along the x-axis, within bulk silicon over a depth of 1 μm. A scheme of the device is given on the left side (diagrams for p-type silicon at equilibrium) and in more detail in Figure B of the Supplementary Information. Three doping level are represented: moderate (p, n; doping of 3 × 10^15^ cm^−3^), high (p^+^, n^+^; 3 × 10^17^ cm^−3^) and very high doping level (p^++^, n^++^; 1 × 10^19^ cm^−3^). In all cases, *E*_*F*_ is set at 0 eV.

The two Schottky diodes Ag/Si and Si/HF are back-to-back. As expected, the space-charge region (SCR) decreases as the doping level increases. At the Ag/p-Si contact, the valence band describes a near-surface peak that may appear unusual in a band diagram. This is actually due to the influence of the Electrolyte/Si junction which has a more pronounced band bending (at equilibrium). It results in a lateral modulation of the band even under the silver contact (at 6 nm from the edge surrounding the contact). This illustrates the advantage of 2D modeling to reveal such lateral effects that cannot be depicted from simplified 1D band diagrams.

To mimic MACE, the oxidizing power of H_2_O_2_ is accounted for by a positive polarization applied to silver vs. electrolyte, as shown in Figure 3 (bottom row) in the case of moderate doping (p, n). The potential drop is distributed spatially between these two junctions according to their electronic characteristics. With n-type silicon, the Ag/n-Si diode is under forward bias while the n-Si/HF diode is blocking. Most of the potential drop occurs at the Ag/Si interface. In the case of p-type silicon, the Ag/p-Si diode is blocking while the p-Si/HF diode is under forward bias and most of the potential drop occurs at the Ag/Si interface as well. Therefore, the systematic presence of a blocking diode should not allow delocalized MACE, i.e., electron transfer from surface silicon atoms (oxidized and dissolved in HF) to H_2_O_2_ molecules (reduced on silver).

Results from the literature show, however, that different silicon doping conditions and enhanced electric field due to geometrical effects (contact size, pore tip) can radically alter the charge transfer for this type of diode under reverse polarization (Lehmann, 2002; Smit et al., 2002, 2004; Donolato, 2004; Vostokov and Shashkin, 2004).

#### N-Type Si/HF Diode

While for n-type silicon under anodic (reverse) polarization in the dark, the absence of h^+^ in the valence band prevents porous silicon formation, a mesoporous silicon layer is formed for n^+^ and n^++^ silicon. The anodic current is actually produced by tunneling of electrons through the SCR from silicon surface atoms to the conduction band. This occurs at relatively low potentials (especially for n^++^) because the electric field at a mesopore tip is much stronger (due to the radius of curvature) than at a flat surface (Lehmann, 2002, chapter 8). This effect becomes significant when the radius of curvature of the pore tip is smaller than the width of the SCR. Regarding MACE, to the best of our knowledge, n-type silicon etching via the conduction band has only been proposed by Yae et al. (2010), in the particular case of palladium particles in HF medium without oxidizing agent. They demonstrated that the oxidation of surface silicon atoms was coupled to the reduction of water on palladium by electron injection into the conduction band.

I-V characteristics established with our n-type silicon samples in HF at the concentration used for MACE (*cf*. Figure D in Supplementary Information), clearly show a lack of anodic current for n-type silicon, a small current for n^+^-type silicon and a significant current for n^++^-type silicon. A porous silicon layer is rapidly built at the surface in the latter case.

**Figure 4:**
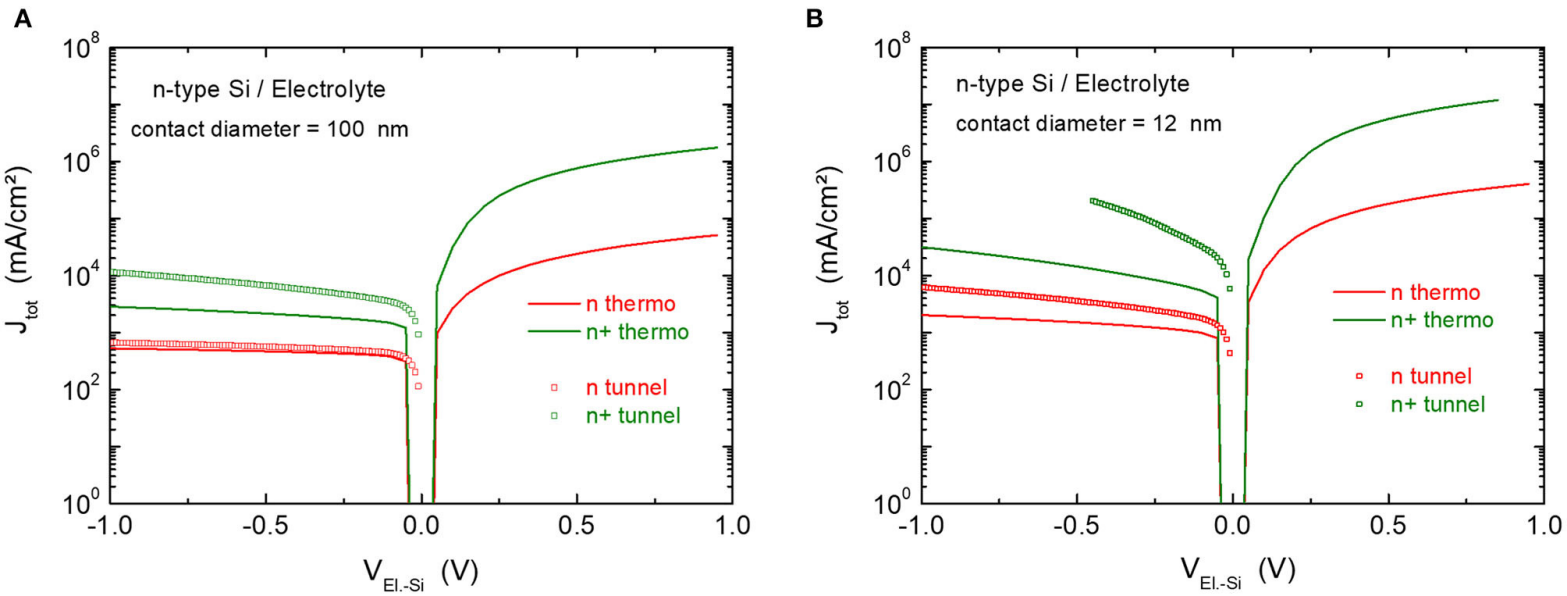
I–V characteristics of n and n^+^ silicon in contact with an HF electrolyte (W = 4.5 eV). The electrolyte contact diameter is 100 nm **(A)** and 12 nm **(B)**. The current is calculated either for thermionic emission (including image potential effect), and with or without tunneling current contribution.

We investigated whether it was possible to retrieve these results by establishing the I-V characteristics from electrostatic modeling (Silvaco) of an electrolytic contact (HF) on heavily, moderately and lightly doped silicon. To evaluate the size effect, circular silver pads with a diameter of 100 and 12 nm were tested (*cf*. Figure C of Supplementary Information). Figure 4 plots the I-V characteristics so obtained. The current is calculated for thermionic emission only (including image potential effect) or with taking into account tunneling transport through the SCR (field emission and thermionic field emission (Sze, 1981; Rhoderick, 1982).

With an electrolytic contact of 100 nm, the contribution of tunneling to the reverse thermionic current is negligible for n-type silicon but becomes significant for n^+^-doped silicon (i.e., it increases by a factor of ~3), as shown in Figure 4A. When the contact diameter is reduced from 100 to 12 nm, the reverse current density increases regardless of the doping level. This is due to a reduced SCR thickness (Smit et al., 2002), but the (thermionic) forward current also increases (not shown in Figure 4B) which indicates a lowering of the barrier height as well (Vostokov and Shashkin, 2004).

The modeling thus makes it possible to account for the size effect in Schottky contacts (higher tunneling current, lower barrier height) that has been highlighted by Smit et al. (2002), Donolato (2004), and Hugelmann and Schindler (2004).

#### P-Type Si/Ag Diode

**Figure 5:**
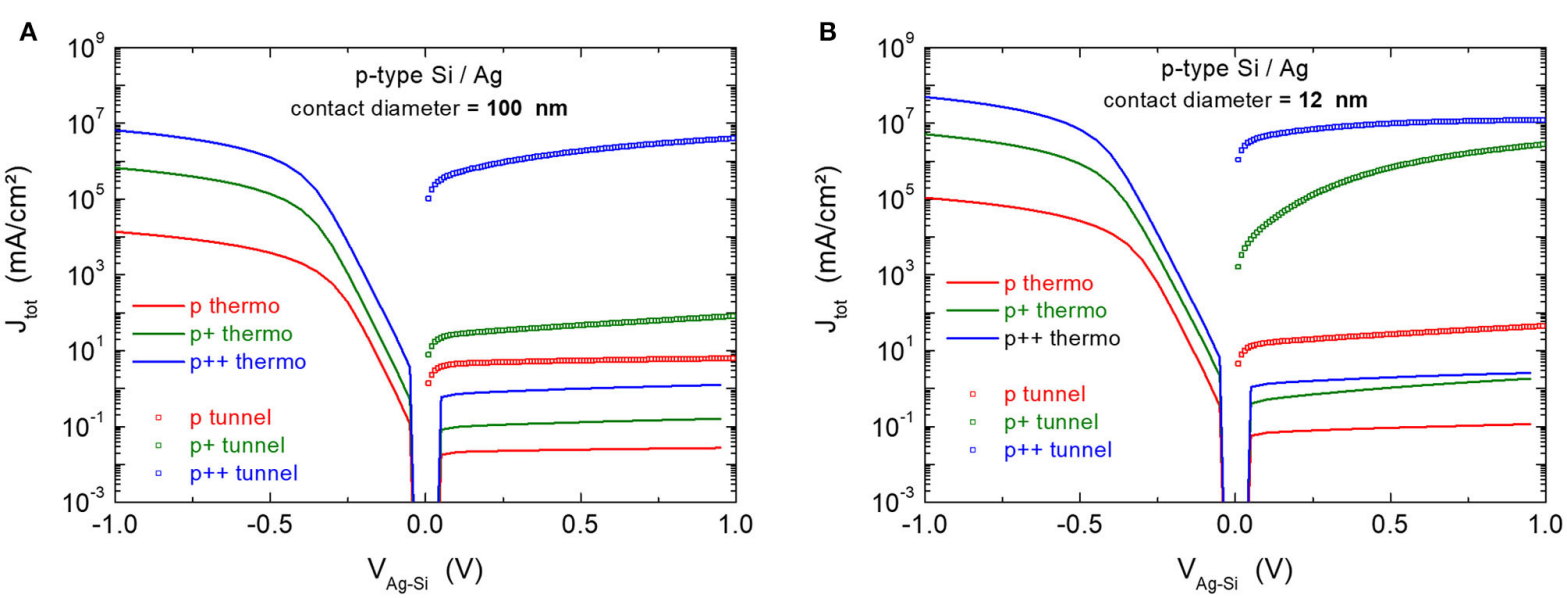
I–V characteristics of p, p^+^ and p^++^ silicon in contact with silver (W = 4.64 eV). The silver contact diameter is 100 nm **(A)** and 12 nm **(B)**. The current is calculated either for thermionic emission (including image potential effect), and with or without tunneling current contribution.

Under anodic polarization, the p-Si/Ag diode is blocking. Figure 5 shows the I-V characteristics of p-Si/Ag diodes established by modeling, for two contact sizes (100 and 12 nm) and three doping levels (p, p^+^ and p^++^).

The reverse-biased Si/Ag solid junction (V_Ag−Si_ > 0) produces a non-negligible current if tunneling is taken into account, even when the doping level is low (p) and the contact size large (100 nm). The current density at +0.5 V is 220 and 360 times higher than that calculated for thermionic emission alone, in the case of p and p^+^-doped silicon, respectively. As a result, the diodes are less rectifying as the silicon doping level increases.

The size effect (reduction in diameter from 100 to 12 nm) is not pronounced for p and p^++^-doped silicon but important for p^+^-doped silicon. In this latter case, the reverse current density (tunneling) is almost equal to the forward current density (thermionic emission) for the 12 nm silver contact. Therefore, both the increase in doping level and the downsizing of Schottky diodes to nanometer sized silver contacts lead to significant tunneling currents.

#### Summary of the Modeling Results

To sum up, it appears that delocalized MACE is not possible with lightly doped p- or n-type silicon since in each case one of the two (back to back) diodes is blocking. Some porous silicon is actually visible at the very top of the pores though (*cf*. Figure 2), probably as a result of a very small current accumulated during the 20 min of etching (the surface experiences the longest etching time). This phenomenon is accentuated by the presence of numerous silver nanoparticles near the surface that etch the substrate laterally (see the large number of pore sections visible in the cross-section images of Figure 2), contributing to a higher leakage current density in this region and thus to some porosification of the surface. In addition, silver oxidation by H_2_O_2_ with diffusion of Ag^+^ in solution far from the initial location can also lead to non-local etching, as studied in detail by Chiappini et al. (2010) and Geyer et al. (2013), and this phenomenon would be more important near the surface because of the high number of silver nanoparticles there.

On the contrary, MACE can be delocalized with heavily doped silicon (n^++^, p^++^), for which the reverse currents are significant. In both cases, only majority carriers are involved. With highly doped silicon (n^+^, p^+^), the situation is intermediate for the MACE conditions used in this work. With p^+^ silicon, we observe the presence of porous silicon all along the main pores, whereas for n^+^ doped silicon, porous silicon only appears at the top of main pores (i.e., for long exposure times to HF), which implies that the reverse current at n^+^-Si/HF exists but is low.

### Control of the Pore Morphology

The experimental and modeling results discussed above acquire a special interest in the fabrication of submicrometric silicon surface textures, whose 3D aspect is a key to control the samples behavior when interacting with light, e.g., in the case of solar cells or photodetectors.

This type of texturization has shown its interest in solar cells with the so-called “black silicon.” Black silicon can be obtained by various techniques, e.g., plasma etching, laser or chemical treatments (Otto et al., 2015). Some studies have shown that MACE with silver nanoparticles followed by a light alkaline etching allows the formation of black silicon for very high efficiency solar cells (Oh et al., 2012). In this case, the pores were limited in depth and width to ~ 250 and 100 nm, respectively, without a well-defined morphology. More generally, and as we have shown, MACE with silver nanoparticles produces straight mesopores over a range of doping going from p^+^ to n^+^. An increase in HF and H_2_O_2_ concentrations or in the relative fraction of H_2_O_2_ *vs*. HF results in straight mesopores as well (Chartier et al., 2008), hence the chemical control of the nanostructure morphology is not possible with silver catalysts.

On the other hand, MACE with other metals gives different results. Gold nanoparticles have also been investigated for MACE applied to solar cell processing (Koynov et al., 2006; Algasinger et al., 2013; Otto et al., 2015). Silicon nanocones are obtained (~ 500 nm in height and 250 nm in width). The resulting optical properties are very good, with a low effective reflectivity (~1%) and efficient light trapping. However, no means of morphology control was reported. MACE with platinum nanoparticles leads to the formation of cone-shaped macropores (after dissolution of porous silicon) with efficient light coupling properties as well (Torralba et al., 2016). Obtaining these structures rather than straight pores with silver is linked to the nature of the Si/Metal contact, ohmic with gold and platinum, Schottky with silver (for silicon doped between p^+^ and n^+^).

However, if the objective is to precisely control the morphology of the etched structures, for instance for the design of specifically texturized devices (Pinna et al., 2019), even with these metals MACE needs to play on additional parameters. In the case of platinum, it was necessary to add an electrochemical polarization in order to adjust the rate of porous silicon formation around the mesopore etched by the nanoparticles and hence to control the opening of the cone-shaped macropores (Torralba et al., 2016). From a practical point of view, the disadvantage of using gold or platinum as catalysts is their high cost and the need to use a strong oxidant (*aqua regia*) to ultimately remove the nanoparticles (at the bottom of the pores) to avoid subsequent contamination or formation of gold/platinum silicide during high temperature treatments.

**Figure 6:**
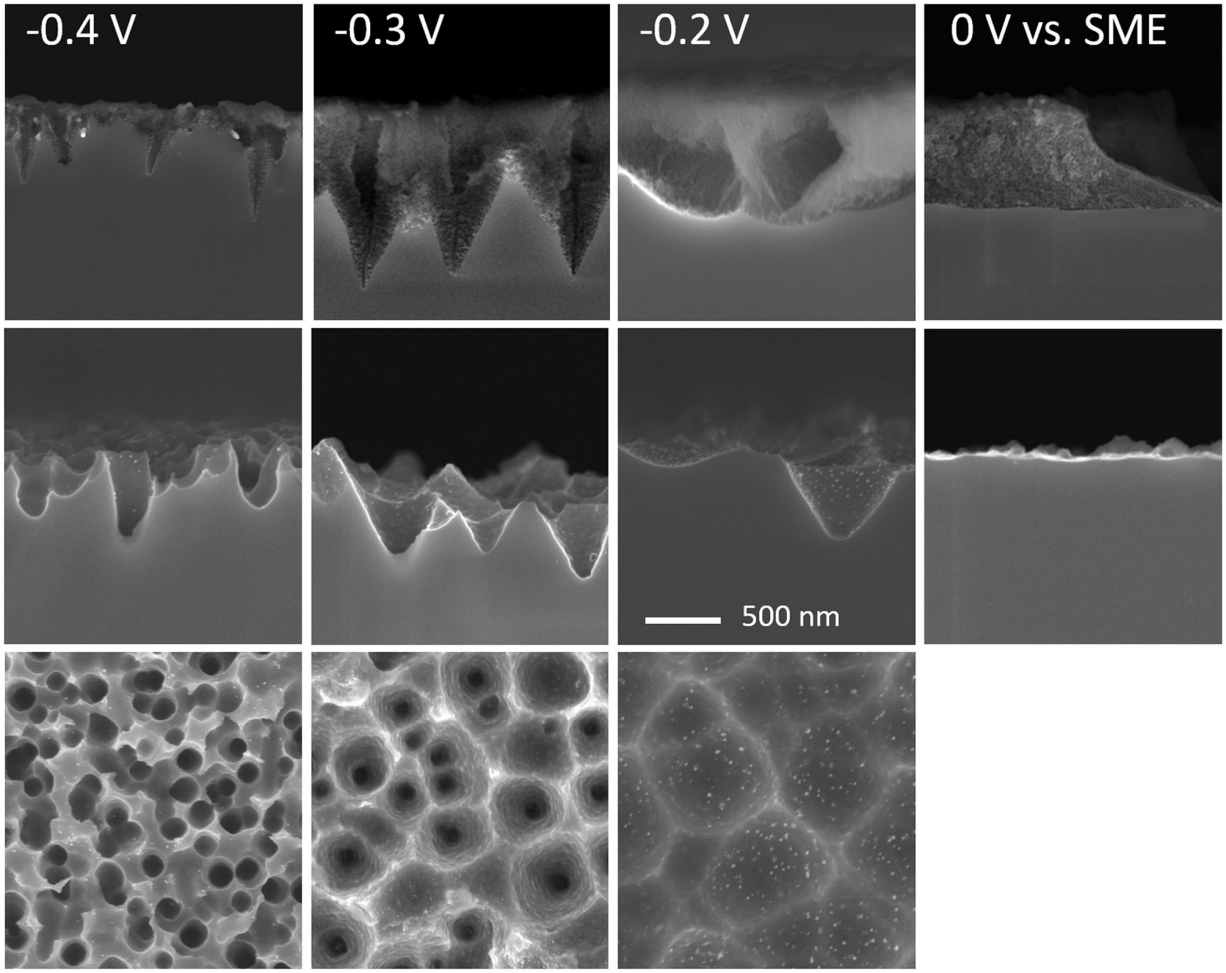
SEM images of cone-shaped pores formed in p-type silicon by MACE with silver nanoparticles in HF-H_2_O_2_ with in addition an external anodic potential. Same magnification for all images. Top row: cross section views after etching. Middle and bottom rows: after etching and porous silicon removal in HF-HNO_3_ in cross section and plane view, respectively. Scale bar: 500 nm.

Taking these problems into account, it would be advantageous to be able to use silver rather than gold or platinum in electrochemically assisted MACE since it is both much easier to dissolve and has a lower cost. For these reasons, a study on MACE of p-type silicon with silver nanoparticles under anodic polarization of the substrate has been carried out. Figure 6 (top line) shows the result of the etching under the same conditions as those of Figures 1, 2 but with the addition of an increasing anodic polarization ranging from +0.1 to +0.5 V. The OCP measured under MACE conditions being of the order of−0.5 V *vs*. SME, this represents potentials ranging from −0.4 to 0 V vs. SME.

In all cases, a porous silicon corolla is formed around the main mesopores, the amount of which increases with the potential. This is expected for p-type silicon under forward bias in HF medium. The anodic current density increases from 1.0 mA cm^−2^ to 6.1 mA cm^−2^ at −0.4 and 0 V vs. SME, respectively.

**Figure 7:**
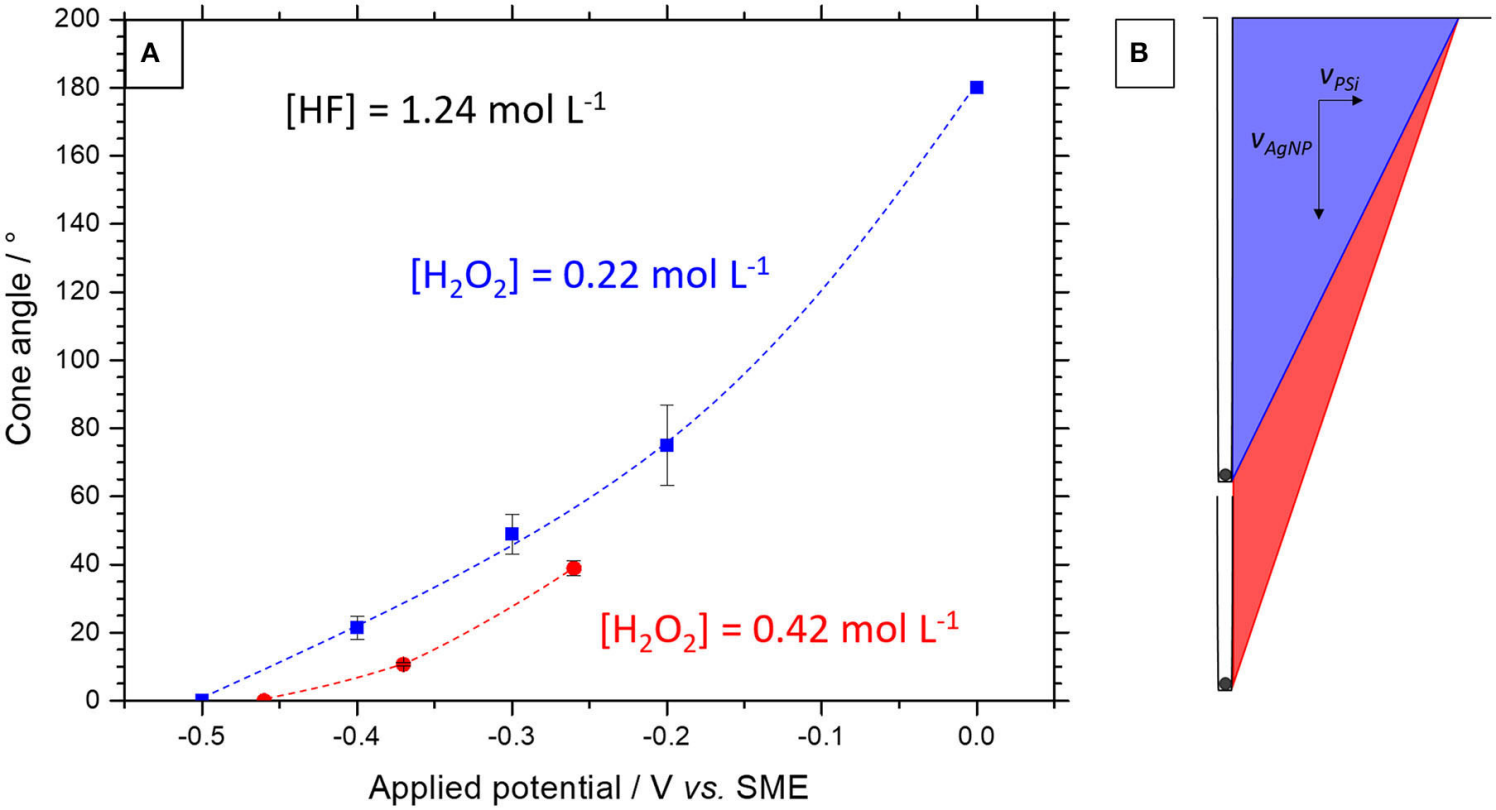
**(A)** Opening angle of the cone-shaped macropores obtained at different anodic potentials during MACE performed in 1.24 mol L^−1^ HF for two different H_2_O_2_ concentrations corresponding to the experiments presented in Figure 6 (0.22 mol L^−1^) **Figure 9** (0.42 mol L^−1^). The error bar represents the standard deviation for about ten measured macropores. The dashed lines are guides for the eyes. **(B)** Scheme of a cone-shaped pore formation by combined MACE and porous silicon anodic formation with etch rates V_AgNP_ and V_PSi_, for low and high H_2_O_2_ concentrations (blue and red half-cones, respectively).

The SEM images of the middle and bottom lines in Figure 6 are obtained after dissolution of the porous silicon layer in HF-HNO_3_ (1:99) in transverse and plan view, respectively. Cone-shaped macropores are then observed with an opening angle that increases as a function of the applied potential, as reported in Figure 7A (blue dashed line and squares, [H_2_O_2_] = 0.22 mol L^−1^).

It should be noted that at −0.2 V vs. SME the angle shown in Figure 7A is 75° (with a relatively large standard deviation) whereas in the corresponding SEM image in Figure 6, very open cones are also observed. Due to the density of the silver nanoparticles, there is a large overlap between the macropores and only those which are by chance isolated develop a porous silicon cone in accordance with the imposed anodic current. In the vast majority of cases, only the base of the macropores remains due to the superposition of the porous silicon cones.

**Figure 8:**
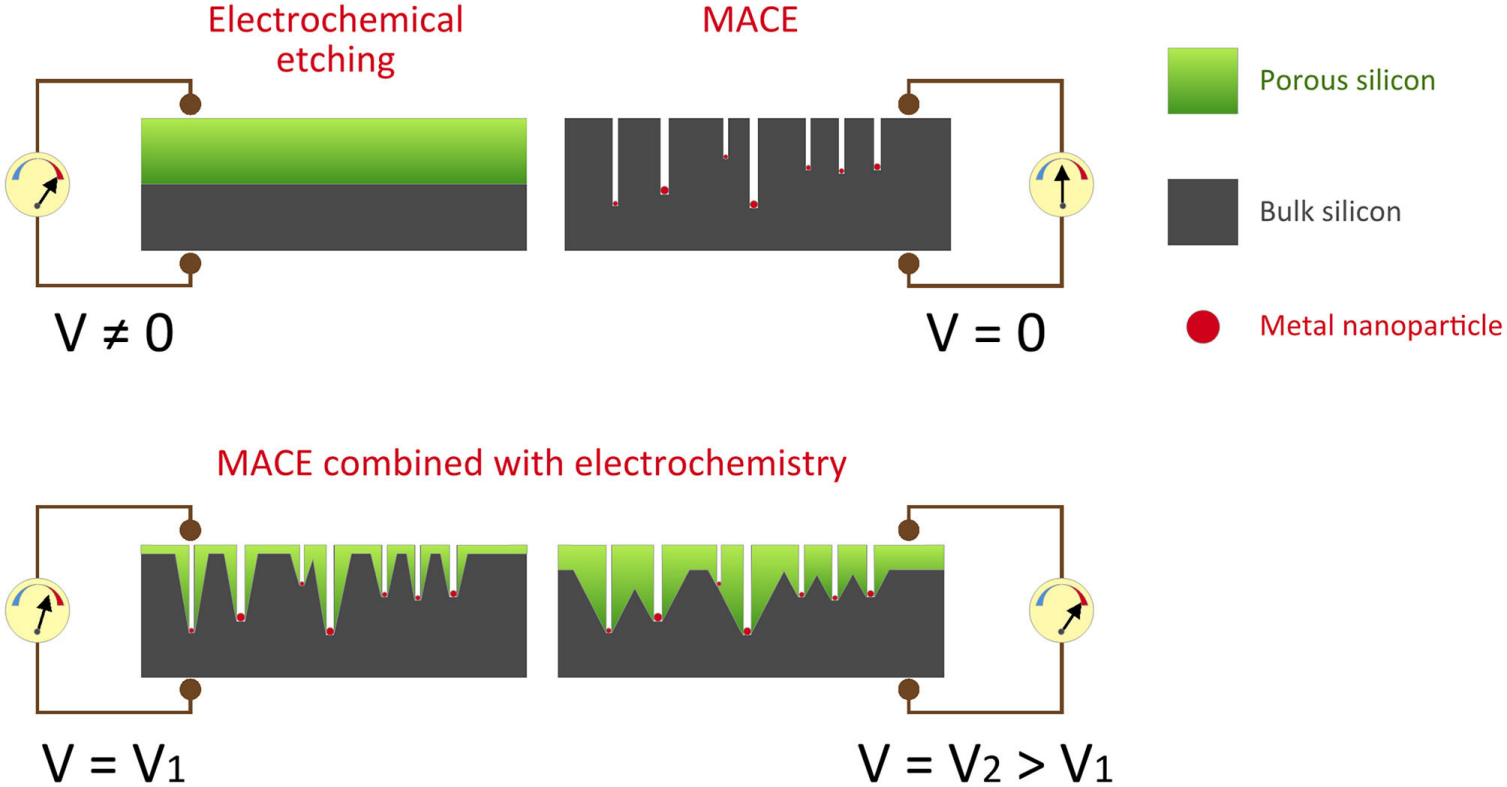
Scheme of the electrochemical anodization and MACE of p-type silicon and their combination for two different applied potentials.

The whole approach is summarized by the diagrams in Figure 8, which brings together the results obtained (on p-type silicon) by electrochemical anodization in HF medium (V_A_ > OCP), by conventional MACE (OCP, V_A_ = 0) and eventually by the two processes carried out simultaneously (MACE at V_A_ > OCP).

To control the macropore dimensions, the applied potential can be adjusted. More opened macropores can be obtained by increasing the anodic potential, as shown for V_2_ > V_1_.

**Figure 9:**
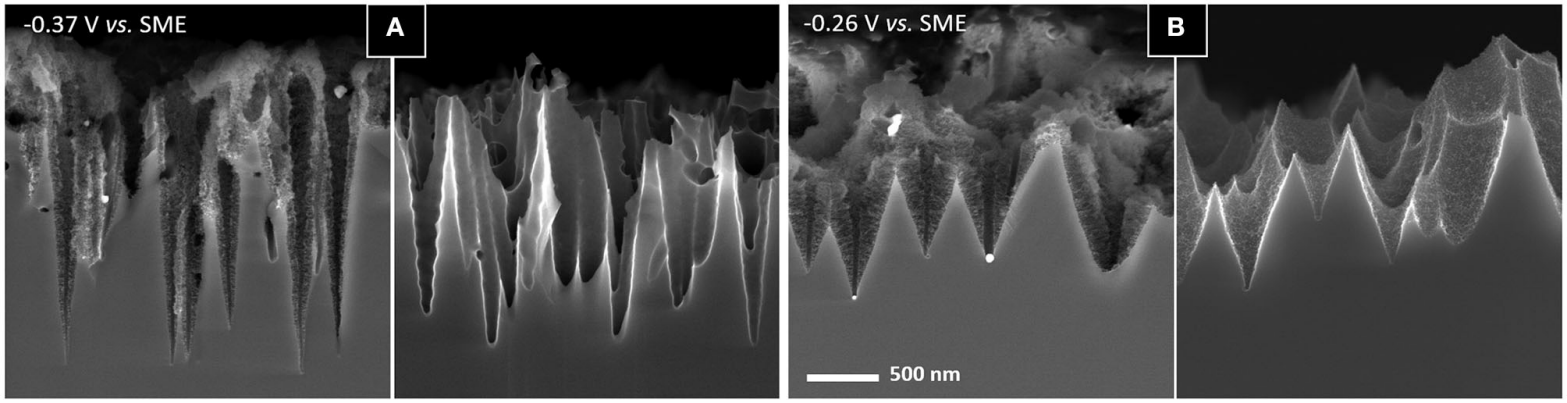
SEM images in cross section of cone shaped pores formed in p-type silicon by MACE with silver nanoparticles in HF-H_2_O_2_ with in addition an external anodic polarization (**A**: −0.37 V vs. SME; **B**: −0.26 V vs. SME) before (left) and after (right) removing porous silicon in HF-HNO_3_. Same magnification for all images.

We have also investigated another option that consist in changing the H_2_O_2_ concentration since it controls the penetration rate of the silver nanoparticles. For that purpose, we tested the effect of doubling the H_2_O_2_ concentration at constant HF concentration. The ρ-value is slightly modified (from 0.85 to 0.75) and the penetration rate is increased by ~50 %. The open circuit potential shifts from −0.50 V to −0.46 V vs. SME when the H_2_O_2_ concentration is increased from 0.22 to 0.42 mol L^−1^. One sample was treated with MACE under anodic polarization at −0.37 V *vs*. SME (+0.1 V *vs*. OCP) and another at −0.26 V vs. SME (+0.2 V vs. OCP). Figure E in Supplementary Information gives the chronopotentiometry of these sample during etching. Figure 9 shows SEM images of the sample surface after these treatments and subsequent etching of the porous silicon layer.

The difference is striking at low applied potential (−0.37 V vs. SME), with cone shaped macropores much more developed and narrower. At −0.26 V vs. SME, the macropore depth with respect to the porous silicon surface is ~1.95 μm (after 20 min).

The angle values of the cone-shaped macropores thus obtained are reported in Figure 7A (red dashed line and dots). Compared to those reported for a lower H_2_O_2_ concentration (blue line and dots), it is clear that, at constant potential, the macropores are ~16–18% narrower. This evolution is logical if we consider that the porous silicon cone shape results from the combination of two perpendicular etchings: (i) the etching normal to the surface of the nanoparticle penetrating the substrate (mesopore) and (ii) the formation of porous silicon normal to this mesopore (i.e., perpendicular to the mesopore wall). This is schemed in Figure 7B. Assuming that the anodic current depends only on the HF concentration, the raise of H_2_O_2_ only increases the penetration rate of nanoparticles (i.e., the mesopore depth). For example, for an applied potential of −0.26 V *vs*. SME, the cone angle increases from 40° to 55° when the H_2_O_2_ concentration is doubled, which would correspond to an increase in the penetration rate of the nanoparticles by a factor of 1.4, in relative agreement with the factor 1.6 measured from the SEM images of Figure 9 (1.9 μm/20 min at −0.26 V vs. SME) and Figure 6 (1.2 μm/20 min at −0.3 V vs. SME). Note that we exclude an influence of the anodic current on the penetration rate of silver nanoparticles due to a preferential collection of h^+^ at the mesopore tips. This remarkable effect has been reported in the literature (Chourou et al., 2010; Huang et al., 2010), but it concerns the electrochemistry in HF medium only (i.e., without H_2_O_2_). In the presence of H_2_O_2_, the lateral curvature of the bands is no longer favorable to the diffusion of h^+^ from p-Si/electrolyte to p-Si/Ag areas.

**Figure 10:**
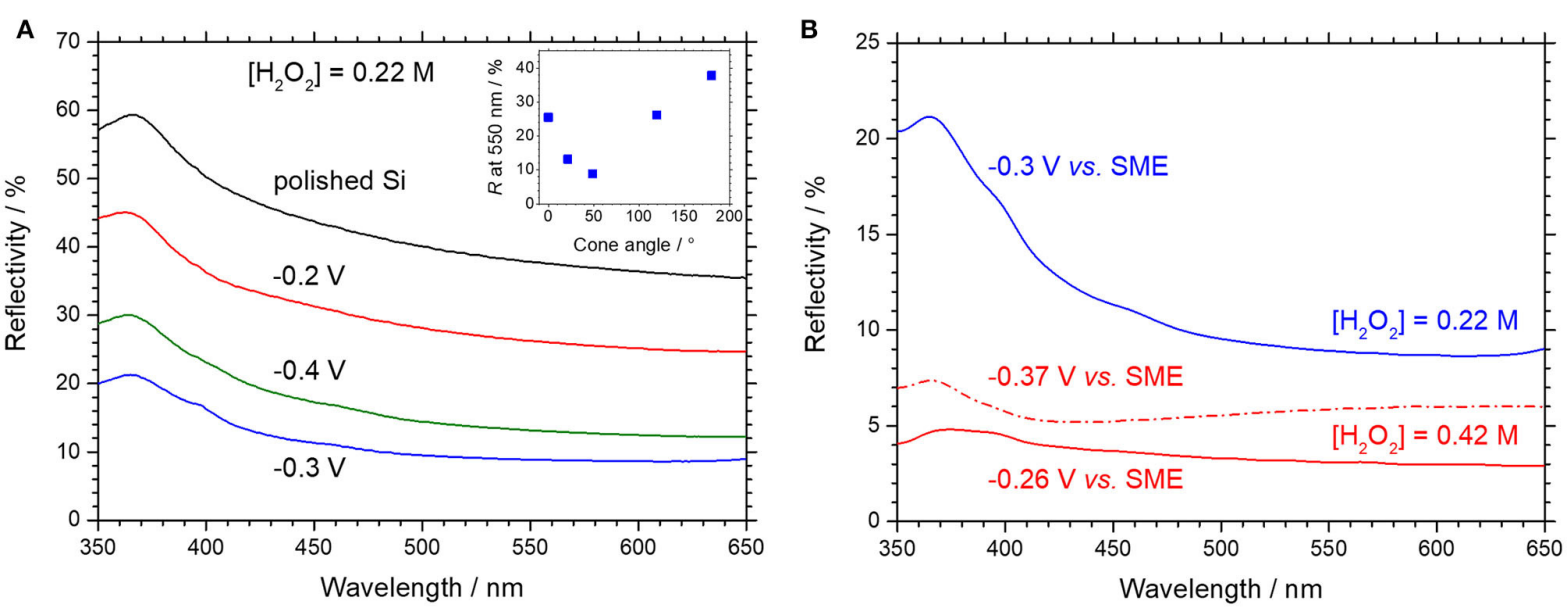
Surface reflectivity of p-type silicon covered with cone shaped macropores obtained by MACE under anodic polarization, followed by dissolution of porous silicon in HF/HNO_3_. MACE is performed in 1.24 mol L^−1^ [HF] and **(A)** 0.22 mol L^−1^ [H_2_O_2_] or **(B)** 0.42 mol L^−1^ [H_2_O_2_]. Anodic potentials applied during MACE are indicated in all cases. The inset of **(A)** gives the reflectivity at 550 nm as a function of the cone angle.

The surface reflectivity was measured from 350 to 650 nm for textured surfaces with cone-shaped macropores of different opening angles, as shown in Figure 10, for a low (A) and high (B) concentration in H_2_O_2_.

The reflectivity spectra in Figure 10A are found to be similar to that of polished silicon, indicating that there is no optical interference due to the presence of an intermediate index layer, as would be the case with remains of porous silicon. The evolution of the reflectivity at 550 nm with the cone angle is given in the inset of Figure 10A. For the sample treated at −0.2 V vs. SME, a high reflectivity is measured, that corresponds to shallow macropores with large openings (*cf*. Figure 6). The least reflective surface (9 % at 550 nm) is obtained for cone-shaped macropores with an opening angle of ~ 49° (*cf*. Figure 6, −0.3 V vs. SME), which is comparable to the reflectivity of (100) oriented silicon with inverted square-based pyramids (Magnin et al., 2014) obtained by lithography and alkaline etching for high efficiency solar cells. At −0.4 V *vs*. SME, the macropores are smaller and narrower and the reflectivity slightly higher (13 %).

These results can be rationalized from the comprehensive analysis of the optical properties of submicrometer structures (black silicon) developed by Otto et al. (2015). The light trapping performances depend mainly on the correlation length *L*_*c*_, which corresponds approximatively to the lateral distance between adjacent peaks and valleys and influences the fraction of scattered light. The larger the correlation length, the better the light trapping. At the same time, the reflectivity also increases with *L*_*c*_ due to backscattering. The second important parameter is the peak to valley height *H* of the structures, which is detrimental to the antireflection properties and light trapping if it is less than ~500 nm, since the refractive index gradient is not sufficient to couple light efficiently.

At low H_2_O_2_ concentration, macropores obtained at −0.4, −0.3 and −0.2 V vs. SME exhibit: *L*_*c*_ = 0.23, 0.32, 0.52 μm, and *H* = 0.28, 0.52 μm, 0.28 μm, respectively. Therefore, the lowest reflectivity is obtained at−0.3 V because the macropores are narrower than those obtained at −0.2 V vs. SME and much deeper than those obtained at −0.4 V vs. SME.

At high H_2_O_2_ concentration, the macropores get deeper and narrower, which translates for etching at −0.37 V and−0.26 V in: *L*_*c*_ = 0.23 μm and 0.34 μm; *H* = 1.49 and 0.87 μm, respectively. The reflectivity spectra of the sample etched at −0.37 V vs. SME does not vary monotonically like the others but exhibit a minimum (5.9 %) around 410 nm (*cf*. Figure 10B). Despite suitable *L*_*c*_ and H values, the reflectivity is not that low, probably because the refractive index profile in the macroporous layer is too steep (*cf*. Figure 9A). The macropores obtained at−0.26 V vs. SME exhibit cone angle of ~ 40°, and a reflectivity of 3% at 550 nm (*H* = 0.87 μm, *L*_*c*_ = 0.34 μm). This number is comparable to some values reported in the literature for black silicon (Oh et al., 2012; Hirsch et al., 2016; Li et al., 2017). However, black silicon obtained by MACE with Au nanoparticles can lead to effective reflectivity as low as ~ 0.6–1 % under certain etching conditions, as reported by Algasinger et al. (2013) and Otto et al. (2015). In this case, the difference lies in structures with a *L*_*c*_ closer to the optimal value of 0.1 μm, together with a significant depth *H* of 0.65 μm. On the other hand, larger *L*_*c*_ values, as obtained here by MACE with silver nanoparticles under anodic polarization, lead to strong light trapping. With this respect, the cone-shaped macropores should be as efficient as the best “black silicon” nanostructures obtained by photoelectrochemical etching that exhibit a *L*_*c*_ value of 0.36 μm (Otto et al., 2015). This can be advantageous in several cases, like for instance in ultrathin silicon solar cells.

## Conclusion

In this work, we studied the mechanisms involved in MACE of silicon in HF-H_2_O_2_ medium using silver nanoparticles with the aim to explain and control the process of 3D surface nanostructuring.

A systematic investigation over the whole range of silicon doping was conducted. We show for the first time that there is a delocalized MACE of p^++^ and n^++^ silicon with formation of a porous silicon cone around the main mesopores. Bibliographic data and 2D modeling have allowed to rationalize the Ag/Si/electrolyte (HF) system into two nanodiodes (Ag/Si and Si/HF) in series. During MACE, H_2_O_2_ induces a positive polarization of silver with respect to the HF electrolyte, with one of the nanojunctions under reverse (blocking) polarization, either Ag/Si for p-type silicon or Si/HF for n-type silicon. Simulations of the I-V curves of these nanodiodes showed that the reverse current increases with the doping level and when the size of the metal contact (i.e., nanoparticle size) decreases, both factors leading to a more important contribution of tunneling current to the overall current (in the case of high dopings, the reverse and forward currents can even be of the same magnitude). Therefore, in the case of n-type silicon, and as already known for the electrochemical formation of mesoporous silicon in the dark, the delocalization of MACE can occur by injection of electrons from silicon surface atoms into the conduction band (majority carrier process). By analogy with these results, the formation of cone-shaped macropores in p-type silicon was obtained with silver nanoparticles and the help of an external anodic polarization during MACE. The anodic current makes it possible to form a porous silicon cone around the mesopores etched by the nanoparticles. We have shown that the angle of the cone and the depth of the macropores can be controlled either by the anodic potential (i.e., the rate of porous silicon formation, parallel to the surface) or through the H_2_O_2_ concentration which determines the penetration rate of the silver nanoparticles (perpendicular to the surface). Black silicon with reflectivity values of about 3% was obtained (after porous silicon removal) with a macropore depth of 0.8 μm and a correlation length of 0.34 μm. Gold or platinum nanoparticles are already known as MACE catalysts leading to the formation of nanocones or cone-shaped pores. As demonstrated here, silver allows a high level of control in nanostructuring but presents also the advantage of being less expensive and more easily removed, a crucial step to avoid contamination during subsequent processing.

## Data Availability Statement

The datasets generated for this study are available on request to the corresponding author.

## Author Contributions

All authors participated in the design and planning of the research. EP and ET participated to the deposition of silver nanoparticles, MACE experiments and SEM analysis. SLG did the modeling and numerical simulations of the Ag/Si/HF system and the nanodiodes. ET and SB performed the electrochemical experiments. All authors participated to the data analysis discussion. SB wrote the first draft of the manuscript with SLG and ET providing feedback. GM and SB wrote the final version of the manuscript.

## Conflict of Interest

The authors declare that the research was conducted in the absence of any commercial or financial relationships that could be construed as a potential conflict of interest.
