# Supplementary material for: Mesopore Formation and Silicon Surface Nanostructuration by Metal-Assisted Chemical Etching With Silver Nanoparticles
Source: Front Chem. 2020 Jul 30;8:658. doi: 10.3389/fchem.2020.00658 (PMC7416550; doi:10.3389/fchem.2020.00658)
Supplement: Supplementary file 1 [file Data_Sheet_1.pdf]

Supplementary Material

This document provides the supplementary information indicated in the manuscript "*Mesopore formation and silicon surface nanostructuration by metal-assisted chemical etching with Ag nanoparticles*" by **E. Pinna, S. Le Gall, E. Torralba, G. Mula, C. Cachet-Vivier, S. Bastide.** Submitted for publication in Frontiers in Chemistry.

Silver nanoparticles deposition

Two deposition methods of silver nanoparticles have been employed. One consisted in sputtering a 10 nm thick silver layer which was then annealed under argon at 275°C for 20 min. The other is a chemical deposition (analogous to MACE) in $AgNO_3$-HF. The result is shown in **Figure** A**A-1** and **A-2**, respectively.

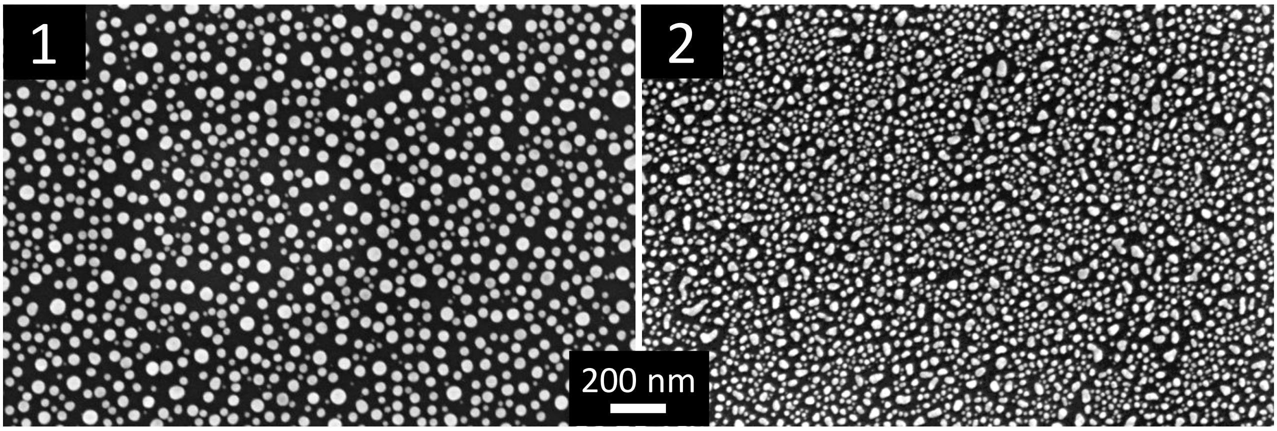

Figure A. Silver nanoparticles on silicon obtained by (1) sputtering of a silver layer (10 nm thick) and annealing under Ar at 275°C during 20 min; (2) chemical deposition in $AgNO_3$-HF (1 and 90 mmol L^{-1}, respectively) during 1 min (RT).

By sputtering, round-shaped nanoparticles with an average (Feret) diameter of 36 ± 11 nm are obtained, at a density of 314 μm^{-2}. By chemical deposition, the silver nanoparticles exhibit various shapes, from elongated to spherical, with an average (Feret) diameter of 26 ± 11 nm and at a density of 817 μm^{-2}. Interestingly, the penetration rate of these silver nanoparticles at OCP in HF-H_2O_2 is different depending of the deposition method: 0.34 µm/min for sputtered silver *vs.* 0.13 µm/min for chemically deposited silver. This is likely due to the 2.6 times lower surface density of nanoparticles in the case of sputtered-annealed silver. As demonstrated in a previous work about MACE of amorphous silicon (Greil et al., 2015), the lower the surface density, the higher the share of H_2O_2 (limiting reactant) for each nanoparticle, hence the higher the penetration rate.

Device structure

The modeled structure used for numerical simulations in 2D of the valence band modulation at the Ag/Si/electrolyte interfaces is schemed in **Figure B** (TCAD software; Atlas from Silvaco). The Si substrate has a thickness of 100 µm and a width of 100 nm. A 12 nm large Ag pad located in the middle is surrounded by two electrolyte contacts (43 nm large). The silver and electrolyte phases are separated by 1 nm of insulating vacuum to allow charge transfer only through the Si/Electrolyte and Si/Ag interfaces. The electrolyte contacts are short-circuited (*i.e.* at same potential).

The simulator solves the physical equations governing the electrostatics (Poisson, electro-neutrality) and the transport of e^- and h^+ (drift-diffusion) self-consistently on a 2D mesh. The work functions of Si are taken at $4.07\ eV < W_{Si} < 4.29\ eV$ depending on the doping level,

W_{Ag} = 4.64 eV and W_{El} = 4.5 eV (determined in our experimental conditions, *cf.* (Torralba et al., 2016) and its supplementary information).

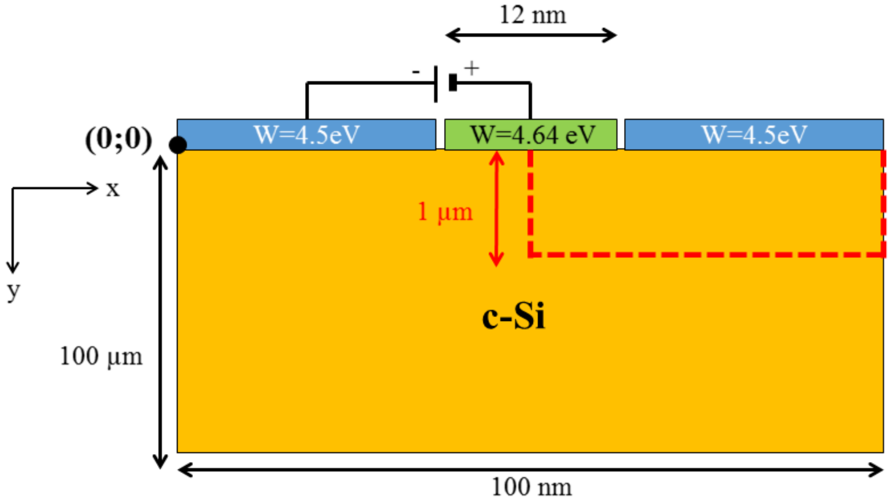

Figure B. Structure used for the band bending modelling of the Ag NP/Si/Electrolyte junction at equilibrium and under an applied polarization mimicking the MACE process. The broken line represents the cutline along which the band bending is plotted in Figure 3 of the article.

The doping concentrations are taken at $3{\times}10^{15}$ cm^{-3} for low doped (n,p), $3{\times}10^{17}$ cm^{-3} for highly doped (n^+, p^+) and $1{\times}10^{19}$ cm^{-3} for heavely doped (n^{++},p^{++}) silicon. The Fermi level is set at 0 eV at the equilibrium. To mimic the MACE process, a positive polarization is applied between silver and the electrolyte. The band diagrams of Figure 3 are measured along the cutline that is displayed by the red dashed line. Note that the scheme is expanded according to *x*. The cutline corresponds to a path of 1 µm from the Ag/Si interface to bulk silicon (in-depth), 50 nm laterally (along the *x*-axis) at constant potential, and 1 µm from bulk silicon to the Si/Electrolyte interface.

Single Schottky diodes, Si/Ag and Si/HF, have been modeled using the device structure shown in **Figure C**. I-V characteristics are simulated for p-type and n-type silicon with three doping levels and two circular contact sizes, 100 and 12 nm in diameter, surrounded by void. The back contact is modeled as ohmic.

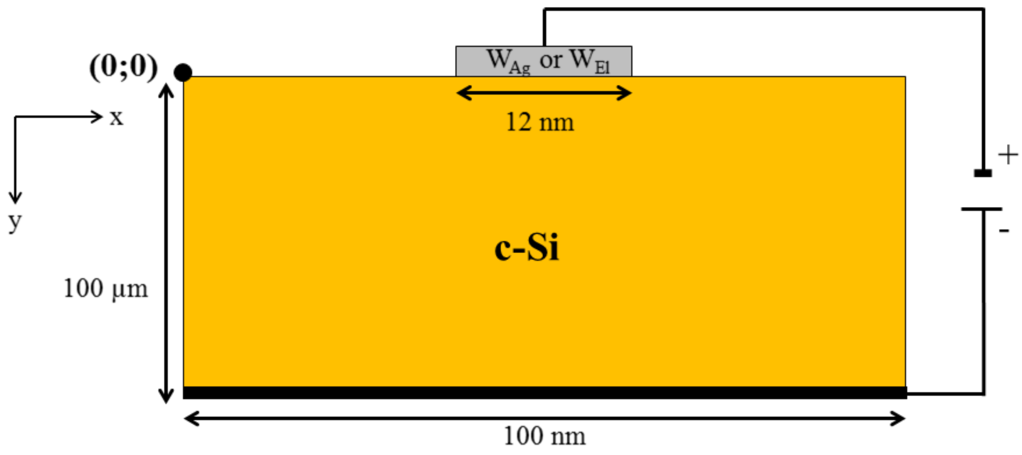

Figure C. Schematic of the structure used for I-V characteristic simulations of Si/Ag and Si/Electrolyte diodes for n and p-type Si of different doping levels. Thermionic currents with and without tunnelling contribution are displayed. The diameters of the silver pad (in green in the image) used in the simulation were 12 and 100 nm.

Calculations of the tunneling current (thermionic field emission and field emission) under reverse bias of the n-Si/HF diode could be performed for n and n^+ doping only. However, the tunneling currents under reverse bias of the Ag/p-Si diode could be calculated for the three doping levels (p, p^+ and p^{++}).

Electrochemistry of n-type Si in HF

Conventional electrochemistry of Si in aqueous HF is performed with the front side of Si in contact with the HF electrolyte (Schottky diode) and a polarization applied on the back side by means of an ohmic contact. A reference and a counter electrode are also present in solution to measure the potential of Si and pass the current, respectively. Note that this is different from MACE experiments, i.e. there is no oxidant in solution (such as H_2O_2) and no metal catalysts on the surface of Si.

I-V characteristics established with our n-type Si samples at the HF concentration used for MACE (*cf.* **Figure D**), clearly show a lack of anodic current for n-Si, a small current for n^+-Si and a significant current for n^{++}-Si. A porous Si layer is rapidly built at the surface in the latter case.

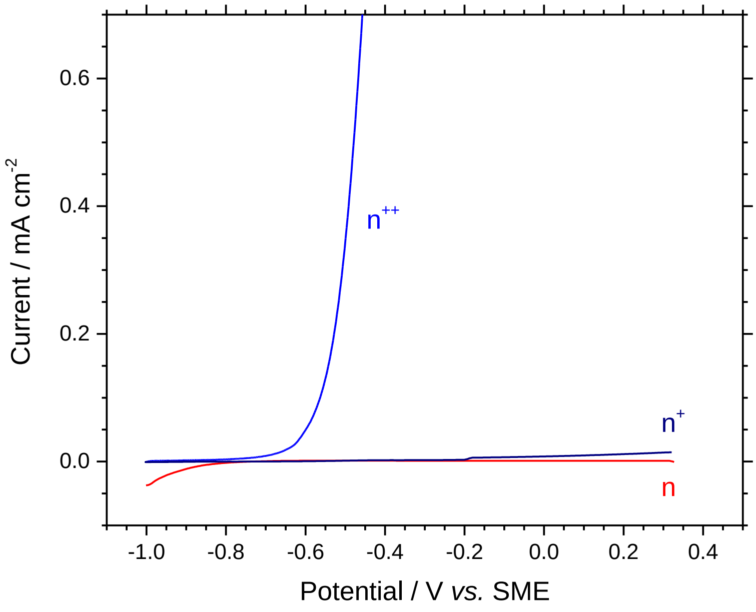

Figure D. I-V characteristics of n, n^+ and n^{++} Si in contact with HF at 1.21 mol L^{-1} (without ethanol). Contact area: 0.5 cm^2. No stirring.

Mesopore morphology

MACE under anodic polarization. Figure E presents the chronopotentiograms of 3 experiments of MACE in HF (1.24 M) and H_2O_2 (0.42 M) media, at OCP and at two different anodic potentials, -0.37 V and -0.26 V *vs.* SME. Each sample (p-Si decorated with silver nanoparticles) is first placed in HF medium. After stabilization, H_2O_2 is added (arrow). The equilibrium potential is consequently shifted to positive values (peak at ~ -0.4 V *vs.* SME), then stabilizes after about 30 seconds around -0.44 to -0.47 V *vs.* SME. Then, an anodic polarization of +0.1 V (-0.37 V *vs.* SME) or +0.2 V (-0.26 V *vs.* SME) is applied for 20 min. The cross-sectional SEM images of these samples are shown in Figure 9 of the article. The average current densities were and 1.8 and 3.4 mA/cm^2 for MACE at -0.37 V and -0.26 V *vs.* SME, respectively.

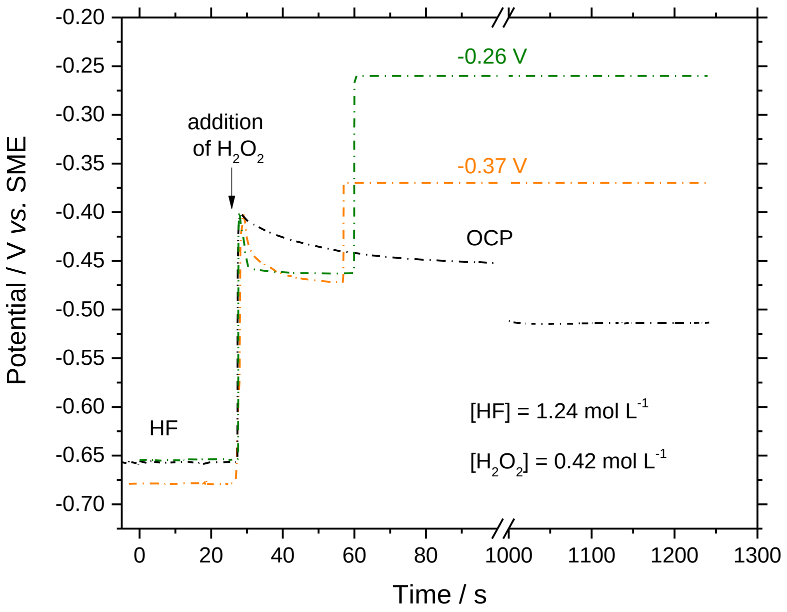

Figure E. Chronopotentiograms of 3 MACE experiments in HF (1.24 M) and H_2O_2 (0.42 M) solution for 20 min, at OCP and at two different anodic potentials, -0.37 V and -0.26 V *vs.* SME. SEM images of the samples are presented in Figure 9 of the article.

References

Greil, S. M., Rappich, J., Korte, L., and Bastide, S. (2015). In Situ PL and SPV Monitored Charge Carrier Injection During Metal Assisted Etching of Intrinsic a-Si Layers on c-Si. *ACS Appl. Mater. Interfaces* 7, 11654–11659. doi:10.1021/acsami.5b02922.

Torralba, E., Le Gall, S., Lachaume, R., Magnin, V., Harari, J., Halbwax, M., et al. (2016). Tunable Surface Structuration of Silicon by Metal Assisted Chemical Etching with Pt Nanoparticles under Electrochemical Bias. *ACS Appl. Mater. Interfaces* 8, 31375–31384. doi:10.1021/acsami.6b09036.
